# *Ixodes scapularis* dystroglycan-like protein promotes *Borrelia burgdorferi* migration from the gut

**DOI:** 10.1007/s00109-015-1365-0

**Published:** 2015-11-23

**Authors:** Jeroen Coumou, Sukanya Narasimhan, Jos J. Trentelman, Alex Wagemakers, Joris Koetsveld, Jasmin I. Ersoz, Anneke Oei, Erol Fikrig, Joppe W. Hovius

**Affiliations:** Center for Experimental and Molecular Medicine, Academic Medical Center, University of Amsterdam, 1105 AZ Amsterdam, The Netherlands; Department of Internal Medicine, Yale University School of Medicine, 06511 New Haven, CT USA; Department of Medical Microbiology, Academic Medical Center, University of Amsterdam, 1105 AZ Amsterdam, The Netherlands

**Keywords:** Tick-borne diseases, Lyme borreliosis, *Ixodes scapularis*, *Borrelia burgdorferi*, Pathogen transmission, Vaccination

## Abstract

**Abstract:**

The causative agent of Lyme borreliosis, *Borrelia burgdorferi*, is transmitted by *Ixodes* ticks. During tick feeding, *B. burgdorferi* migrates from the tick gut to the salivary glands from where transmission to the host occurs. *B. burgdorferi*-interacting tick proteins might serve as vaccine targets to thwart *B. burgdorferi* transmission. A previous screening for *B. burgdorferi*-interacting *Ixodes scapularis* gut proteins identified an *I. scapularis* putative dystroglycan protein (ISCW015049). Here, we describe the ISCW015049’s protein structure and its cellular location in the tick gut in relation to *B. burgdorferi* migration. Secondly, in vivo *B. burgdorferi*–tick attachment murine models were performed to study the role of ISCW015049 during *B. burgdorferi* migration and transmission. In silico analysis confirmed that ISCW015049 is similar to dystroglycan and was named *I. scapularis* dystroglycan-like protein (ISDLP). Confocal microscopy of gut tissue showed that ISDLP is expressed on the surface of gut cells, is upregulated during tick feeding, and is expressed significantly higher in infected ticks compared to uninfected ticks. Inhibition of ISDLP by RNA interference (RNAi) resulted in lower *B. burgdorferi* transmission to mice. In conclusion, we have identified a dystroglycan-like protein in *I. scapularis* gut that can bind to *B. burgdorferi* and promotes *B. burgdorferi* migration from the tick gut.

**Key messages:**

- *B. burgdorferi* exploits tick proteins to orchestrate its transmission to the host.
- *B. burgdorferi* is able bind to an *I. scapularis* dystroglycan-like protein (ISDLP).
- Inhibition of ISDLP in ticks results in lower *B. burgdorferi* transmission to mice.
- ISDLP is a potential target to prevent Lyme borreliosis.

**Electronic supplementary material:**

The online version of this article (doi:10.1007/s00109-015-1365-0) contains supplementary material, which is available to authorized users.

## Introduction

In the USA, *Ixodes scapularis* is the vector of *Borrelia burgdorferi*, the causative agent of Lyme borreliosis [1, 2]. *B. burgdorferi* colonization of the tick gut can occur when uninfected *Ixodes* larvae acquire *B. burgdorferi* when feeding on a *B. burgdorferi*-infected animal [3]. *B. burgdorferi* anchors itself to the tick gut wall by expressing outer surface protein A (OspA), which binds to the tick receptor OspA (TROSPA) [4]. When a *B. burgdorferi*-infected *I. scapularis* nymph feeds on a vertebrate host, *B. burgdorferi* becomes metabolically active, changes its outer surface proteins, and migrates from the tick gut to the salivary glands [5]. Motility of *B. burgdorferi* appears not to be essential for exiting the gut, as described in a newly proposed model called “adherence-mediated migration” by Dunham-Ems et al. [6]. Dunham-Ems and colleagues observed that during tick feeding, *B. burgdorferi* spirochetes initially replicate in the lumen of the gut and remain non-motile. After approximately 24 h, *B. burgdorferi* spirochetes transition into aggregates at the basal lamina of the gut. From here, a small percentage of *B. burgdorferi* penetrate the gut, followed by migration via the hemolymph to the salivary glands into the skin of the host [6].

The close interaction of *B. burgdorferi* with tick gut epithelial cells suggests potential interactions between the tick gut proteins and *B. burgdorferi* proteins that might be critical for *B. burgdorferi* growth in the tick gut and its egress from the gut—a critical step for successful transmission to the vertebrate host. *B. burgdorferi*-interacting tick gut proteins might thus be a vaccine targeted to prevent spirochete migration from the gut and preempt transmission. The advantage of using tick gut proteins as anti-tick vaccines is that migration of *B. burgdorferi* can be targeted early in tick feeding—even before spirochetes have been transmitted to the host. Of note, a vaccine against Lyme disease is currently not available for humans [7]. Since the last decade, tick salivary gland proteins and tick gut proteins have become a target of vaccine development as the tick plays a central role in *B. burgdorferi* transmission [8]. Immunization against salivary gland proteins introduced into the skin that facilitate tick feeding provided (partial) protection against *B. burgdorferi* transmission [9–11] as well as immunization against tick gut proteins [4, 12]. One limitation of vaccine targeting tick gut proteins is that gut proteins might not provide an anamnestic response, since they are not presented to the host during a tick bite. Nonetheless, the future development of cocktail vaccines combining tick gut proteins that facilitate spirochete migration from the gut and salivary antigens that facilitate survival at the bite site might provide a robust impairment of *B. burgdorferi* transmission by simultaneously targeting spirochete egress from the gut and survival at the bite site. Recently, we used a yeast surface display (YSD) approach to screen for *B. burgdorferi*-interacting tick gut proteins and identified three putative *B. burgdorferi*-interacting gut proteins: ISCW008121, ISCW015049, and ISCW015135 [12]. One of them, ISCW008121, was shown to be a transmembrane fibronectin domain-containing *I. scapularis* protein that enabled *B. burgdorferi* adherence to the basal lamina of the gut to facilitate transmission [12]. In this study, we report the characterization of ISCW015049 and examine its vivo role in the context of *B. burgdorferi* transmission and assess its ability to serve as a transmission-thwarting vaccine.

## Methods

### Animal experiments

The rabbit immunized against recombinant *I. scapularis* dystroglycan-like protein (rISDLP) and mice used in the RNA interference (RNAi) experiments were housed and handled under the Guide for the Care and Use of Laboratory Animals of the National Institutes of Health. The animal experimental protocol was approved by the Yale University’s Institutional Animal Care and Use Committee (protocol number 2008-07941, approval date: 31 March 2014). All animal infection experiments were performed in a biosafety level 2 animal facility, according to the regulations of Yale University. In addition, the mice that were used for immunization experiments were housed and handled under the approval of the Animal Care and Use Committee of the University of Amsterdam (DIX103179).

### Ticks

*I. scapularis* nymphs and larvae were obtained from a tick colony at the Connecticut Agricultural Experiment Station in New Haven CT, USA, and ticks were maintained as described earlier [13]. Ticks were allowed to feed to repletion and RNA isolated from guts and salivary glands using TRIzol (Invitrogen, CA, USA) as described earlier [22]. Complementary DNA (cDNA) was synthesized using the iScript RT-PCR kit (Bio-Rad, CA, USA) and analyzed by quantitative PCR for the expression of tick actin and *B. burgdorferi flab* using gene-specific primers (Online Resource 1) and the iQ SYBR Green Supermix (Bio-Rad, Hercules, CA, USA).

### Identification of the full-length transcript of ISCW015049 and purification of rISDLP

First-strand cDNA was synthesized from the total *I. scapularis* gut RNA using a 3′-RACE adapter. The RLM-RACE kit was used to identify the sequence at the 3′-end and 5′-end according to the manufacturer’s instructions (Invitrogen, CA, USA). The identification of the sequence at the 5′-end was performed using ISDLP-specific primers (Online Resource 1). The full-length sequence was assembled using the Web-based software SMART [10] (http://smart.embl-heidelberg.de). Purification of rISDLP was performed as described previously [8].

### ELISA assessment of rISDLP binding to *B. burgdorferi* membrane extract

*B. burgdorferi* membrane extract purified as described previously [12] was coated (1 μg/ml) on high binding microtiter plates (Microlon, Greiner, Germany) overnight at room temperature (RT). Wells were blocked with PBS/1 % bovine serum albumin (BSA) at RT for 1 h and incubated with rISDLP or rTSLPI (3–100 pmol/ml) diluted in PBS/0.05 % Tween 20/1 % BSA for 1 h. Wells were washed and incubated with 1:5000 diluted mouse anti-V5 HRP IgG. Bound antibody was detected using TMB as substrate (Thermo Scientific, IL, USA).

### Confocal microscopy

Confocal microscopy to detect native ISDLP was performed as previously described [12]. Briefly, guts from nymphal ticks (*B. burgdorferi*-infected or uninfected) were dissected and fixed in 4 % paraformaldehyde (PFA). Washed guts were incubated with rabbit anti-rISDLP antibody and bound antibodies detected using fluorescein isothiocyanate (FITC)-labeled affinity purified goat anti-rabbit IgG antibody (Sigma, MO, USA) and nuclei stained with propidium iodide or with TO-PRO-3 iodide (Invitrogen, CA, USA). Control guts were incubated with IgG purified from rabbit anti-ovalbumin sera. Stained guts were visualized under a Zeiss LSM 510 Confocal microscope.

### Pixel intensity quantification

Pixel intensities in the tetramethylrhodamine (TRITC) channel (as a measure of anti-rISDLP serum binding to tick gut ISDLP) or in the FITC channel of confocal images were quantified using the ImageJ 1.47t software. Confocal images of four individual guts were examined in each control and experimental group, and mean pixel intensities representing the average intensity of pixels in the region of interest were obtained in five different regions of each tick gut.

### Immunization of rabbits and mice against ISDLP

Four- to six-week old New Zealand white rabbits were immunized subcutaneously with 30 μg of rISDLP or ovalbumin in complete Freund’s adjuvant (CFA) and boosted twice with 30 μg of rISDLP or ovalbumin at weeks 3 and 6 in incomplete Freund’s adjuvant (IFA). Test bleeds were obtained from ear veins 2 weeks after the final boost, and reactivity to recombinant rISDLP and ovalbumin was assessed by Western blot. Rabbits were euthanized, and serum was obtained by cardiac puncture. Polyclonal IgG was purified from the sera using the Melon Gel IgG purification kit (Thermo Scientific, IL, USA).

For immunization of mice, animals were immunized with 10 μg of rISDLP or ovalbumin in CFA and boosted twice with 10 μg of rISDLP or ovalbumin at weeks 2 and 4 in IFA. To address the role of rISDLP in *B. burgdorferi* transmission, eight *B. burgdorferi* N40-infected nymphs were placed on each immunized mouse. Nymphs were allowed to feed to repletion. Salivary glands and guts were dissected and combined in pools of two to three ticks for quantitative reverse transcription polymerase chain reaction (RT-PCR) as described earlier [9]. DNA was isolated from skin punch biopsies at 7, 14, and 21 days and from heart and joints 21 days post-tick detachment, and *Borrelia* burden was assessed by quantitative PCR as described [9].

### RNAi silencing of *isdlp* in *B. burgdorferi*-infected *I. scapularis* nymphs

RNAi silencing of *isdlp* in ticks was performed as described before [9] using primers specific for *isdlp* with a T7 promoter sequence (Online Resource 1). Double-stranded (ds) *isdlp* double-stranded RNA (dsRNA) was synthesized using the MEGAscript RNAi kit (Ambion/Invitrogen, CA, USA). ds *isdlp* RNA or ds *gfp* RNA (5 nl, 3 × 10^12^ molecules/ml) was injected into the anal pore of *Borrelia*-infected nymphs as described earlier [9]. dsRNA-injected ticks were allowed to feed until repletion and weighed to assess feeding efficiency, and guts and salivary glands were dissected for messenger RNA (mRNA) isolation and quantitative RT-PCR as described above. *B. burgdorferi* burden in mice was assessed by quantitative PCR as described earlier [9].

### Statistical analysis

The significance of the difference between the mean values of the groups was analyzed using a non-parametric two-tailed Mann–Whitney test or a two-tailed Student’s *t* test with the Prism 5.0 software (GraphPad Software, San Diego, CA, USA), and *p* ≤ 0.05 was considered significant.

### Graphics

Figures have been created using the Prism 5.0 software (GraphPad Software, San Diego, CA, USA) and Adobe Illustrator (San Jose, CA, USA).

## Results

### Full-length ISCW015049 encodes a potential transmembrane dystroglycan-like protein

**Fig. 1:**
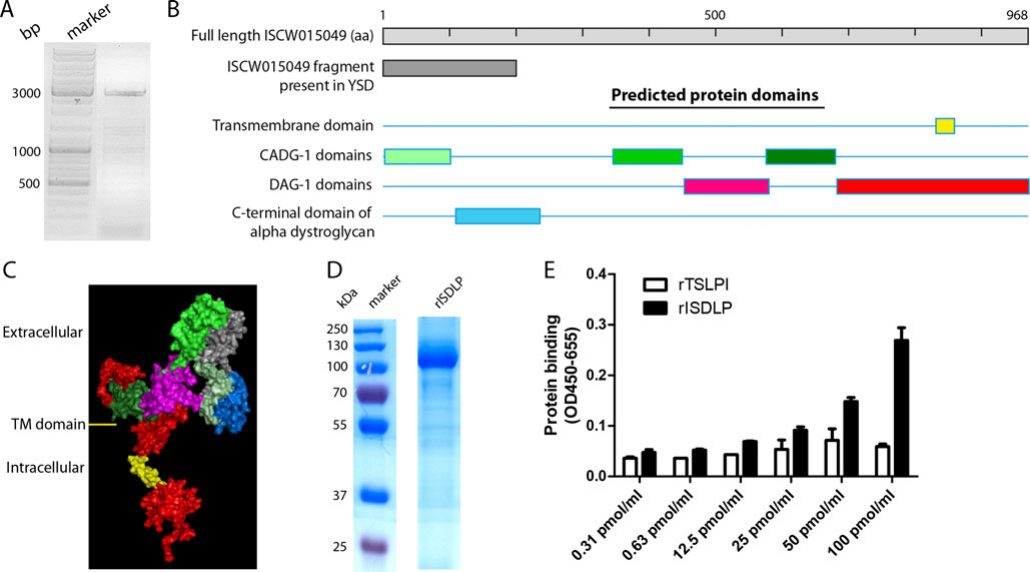
Full-length sequence of ISDLP (GenBank accession number KR782315). **a** Guts from three fed *I. scapularis* were dissected, and RNA was extracted. Reverse transcription polymerase chain reaction (RT-PCR) was performed using primers based on the 3′-end and 5′-end of ISCW015049 which were identified using the 3′-end and 5′-end RLM-RACE. The complete sequence was identified by sequencing of three contigs (1–604, 472–1575, and 1272–2904 bp), which were cloned into the PGEM-T Easy Vector. **b** Protein domains were predicted by SMART domain prediction (http://smart.embl-heidelberg.de) [25], TMHMM v2.0 software, and the conserved domain database of NCBI. If available, *E* values are provided for each domain in parentheses. A potential transmembrane domain was found at aa 828–853; three CADG-1 domains at aa 4–102 (7.63^−3^), 346–450 (3.47^−10^), and 576–680 (2.01^−6^); and two DAG-1 domains at aa 454–588 (2.20^−17^) and 683–968 (7.30^−70^); and a C-terminal domain of α-dystroglycan that was identified at aa 111–236. **c** A 3D model of ISDLP was created using the Phyre2 software (http://www.sbg.bio.ic.ac.uk/phyre2) [26]. Six hundred seventy-six residues (70 %) were modeled at >90 % accuracy. **d** Purified *Drosophila*-expressed recombinant full-length ISDLP electrophoresed on SDS 10 % polyacrylamide gel and stained with Coomassie blue. **e** ELISA assessment of dose-dependent binding of ISDLP to *B. burgdorferi* membrane protein extract-coated plates compared to rTSLPI, a tick protein that is known not to bind to *B. burgdorferi*

The protein sequence present in the YSD screening yeast colony “Clone 3”-matched amino acids (aa) 1 to 201 of the protein annotated on VectorBase as “putative dystroglycan, ISCW015049.” The complete sequence of ISCW015049 was confirmed from the *I. scapularis* gut extract using 3′-end and 5′-end RLM-RACE. Using mRNA from guts of fed *I. scapularis* nymphs, the start and stop codons of ISCW015049 were identified and the complete transcript of 2904 bp was sequenced (Fig. 1a). Identification of the full sequence revealed that the full length of ISCW015049 was 968 aa long, since one fragment of ISCW015049 that annotated as an intron (www.vectorbase.org) was actually found to be part of the ISCW015049 transcript. In silico analysis showed that full-length ISCW015049 has a potential transmembrane domain and is similar to dystroglycan, a widely distributed protein involved in the linkage between the extracellular matrix and the cytoskeleton [14]. The protein sequence of full-length ISCW015049 was 27.0, 28.7, and 26.7 % identical to *Drosophila melanogaster* dystroglycan (NP_725523.3), *Homo sapiens* dystroglycan (AA81779.1), and *Mus musculus* dystroglycan (NP_001263423.1), respectively. Online programs for protein modeling (SMART) predicted that ISCW015049 has three dystroglycan-type cadherin-like (CADG) domains and two dystrophin-associated glycoprotein 1 (DAG1) domains (Fig. 1b). The 201 aa region identified in the YSD screen was predicted to be located on the extracellular region, which consists of one CADG domain and a C-terminal domain of α-dystroglycan (Fig. 1b, c). Based on the similarity with dystroglycan, full-length ISCW015049 is henceforth referred to as the *I. scapularis* dystroglycan like protein (ISDLP) and has been submitted to GenBank (accession number KR782315).

### Production of recombinant ISDLP and confirmation of binding to *B. burgdorferi*

We expressed the full-length protein transcript of ISDLP in a *D. melanogaster* expression system. Binding of purified recombinant ISDLP (rISDLP) (Fig. 1d) to *B. burgdorferi* was confirmed by an ELISA-based binding assay. We observed a dose-dependent increase of rISDLP binding to *B. burgdorferi* membrane extract compared to the control tick protein rTSLPI (Fig. 1e).

### Localization of native ISDLP in the tick gut by confocal microscopy

**Fig. 2:**
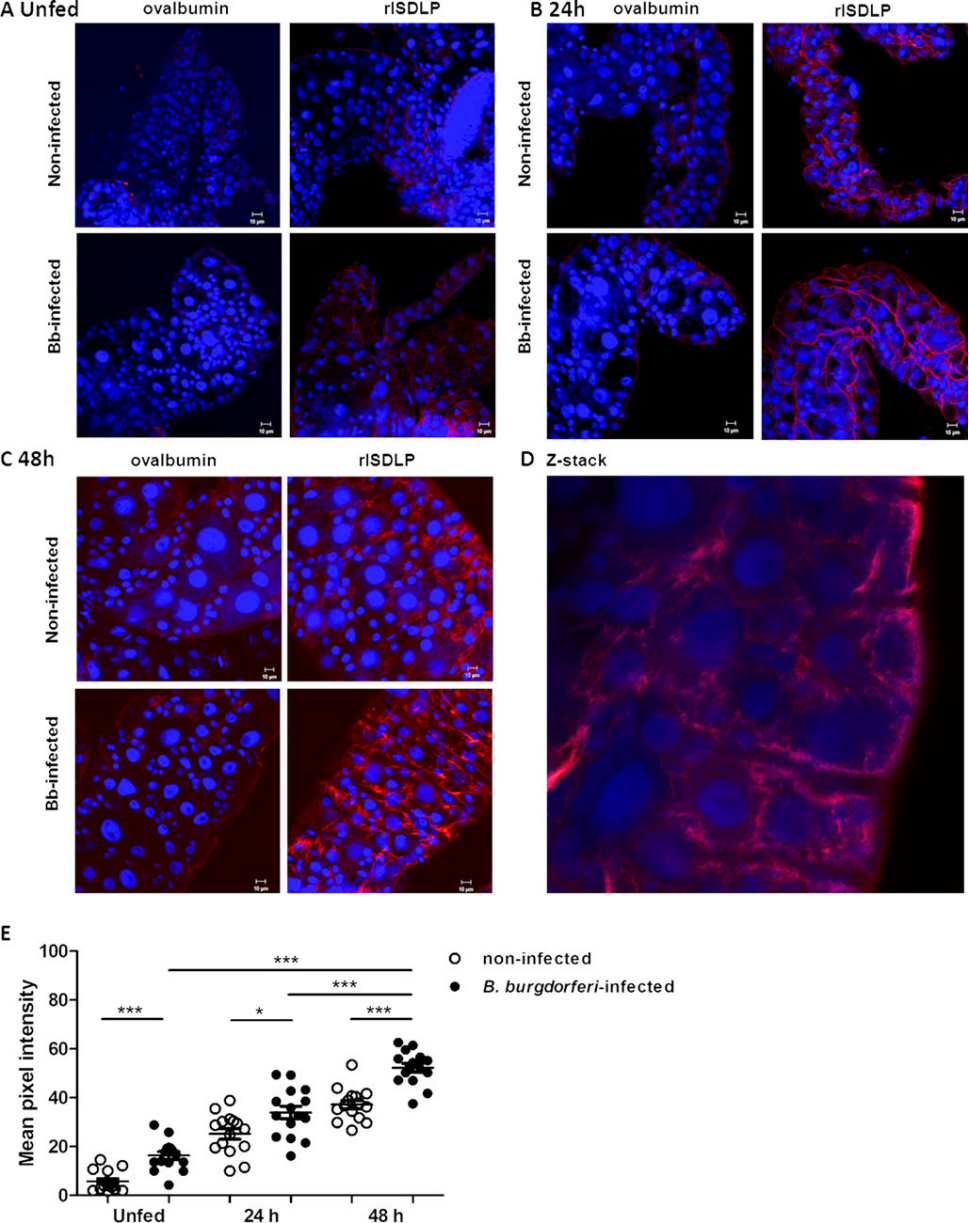
ISDLP is a membrane-bound protein increasingly expressed in the gut during tick feeding. Gut nuclei and ISDLP were stained with TO-PRO-3 (*blue*) and anti-rISDLP rabbit serum (TRITC, *red*), respectively. **a**–**c** Confocal microscopy of guts of unfed (24 and 48 h) and fed uninfected and *B. burgdorferi*-infected *I. scapularis* nymphs. Magnification ×20. Guts stained with anti-ovalbumin IgG (TRITC, *red*) served as antibody control. **d** A Z-stack (magnification ×63) of a *B. burgdorferi*-infected gut after 48 h of feeding gut. **e** Mean pixel intensities of regions of interest in the TRITC channel (representing anti-rISDLP rabbit serum binding to ISDLP) of the confocal images obtained in **a**–**c**, as measured by the ImageJ software. Each *data point* represents one region of interest. The *error bars* represent mean ± SEM, and the mean values significantly different in a two-tailed non-parametric Mann–Whitney test are indicated by an *asterisk* (*p* < 0.05) or by three *asterisks* (*p* < 0.0001)

To study the expression and protein localization of native ISDLP, we generated antibodies against rISDLP by immunizing a rabbit with rISDLP. Unfed and partially fed (24 and 48 h) *B. burgdorferi* and uninfected *I. scapularis* guts were collected, fixed with PFA, and probed with anti-ISDLP rabbit serum or anti-ova rabbit serum as a control. Binding of antibodies in the gut was visualized with immunofluorescence confocal microscopy. Comparing the binding of anti-rISDLP antibodies at the different time points by mean pixel intensity using the ImageJ software showed that ISDLP expression increases significantly during tick feeding (Fig. 2a–c, e). Furthermore, increased binding was observed in *B. burgdorferi*-infected ticks compared to uninfected ticks. In line with our computer-based modeling of ISDLP (Fig. 1), Z-stack imaging suggested that ISDLP is represented both on the cell surface and in the cytosol (Fig. 2d).

### Immunization against rISDLP does not prevent *B. burgdorferi* transmission

**Fig. 3:**
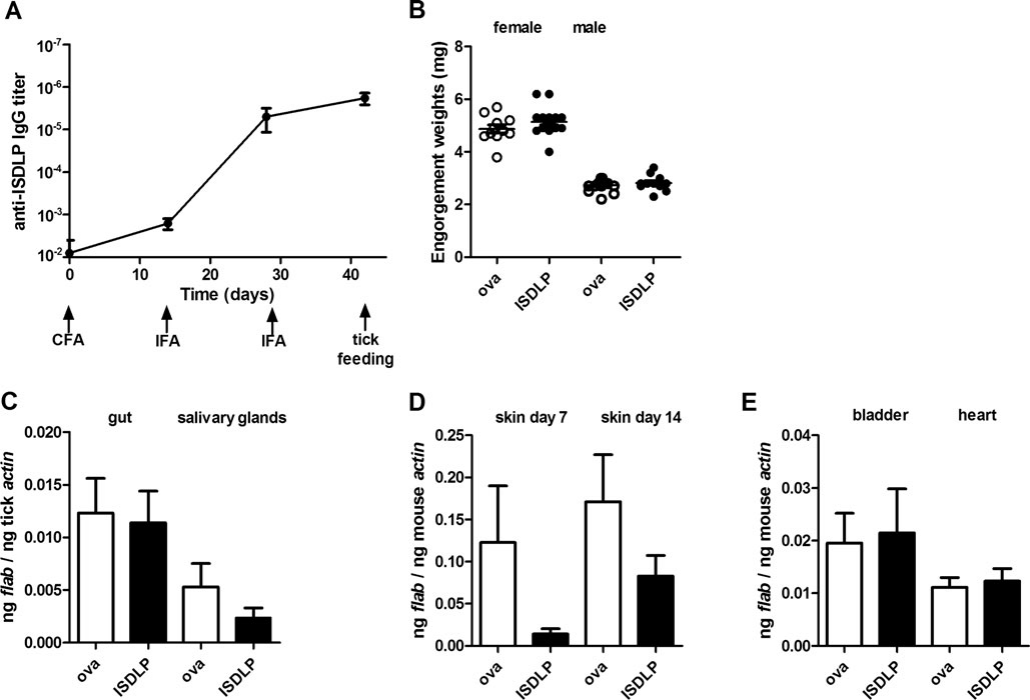
Immunization against ISDLP does not have an effect on *B. burgdorferi* transmission to murine skin. Mice were actively immunized with rIxofin3D-PF or ovalbumin. Eight *B. burgdorferi* N40 ticks/mouse were placed and fed until repletion. Mice were sacrificed after 14 days of *B. burgdorferi* infection. **a** Mean IgG titer in the serum from animals vaccinated against rISDLP, diluted to 1:10^2^ to 1:10^7^ on ELISA-coated plates with rISDLP. The *error bars* represent mean ± SEM. The cutoff for titer was calculated as an OD value of ova-immune serum + 3 SD. **b** Engorgement weights of ticks post-feeding. Each *data point* represents one tick. A tick was considered female when >3.5 mg. **c** RT-qPCR assessment of *B. burgdorferi* burden in tick guts and salivary glands. **d**, **e** qPCR assessment of *B. burgdorferi* burden in the murine skin at 7 days and in the skin, bladder, and heart at 14 days post-tick feeding. The experiment has been performed once with eight mice per group

To test whether immunization against rISDLP would prevent *B. burgdorferi* transmission, we immunized eight mice against rISDLP and eight mice against ovalbumin. We achieved good IgG titer levels against rISDLP in the sera of mice that were immunized against rISDLP after immunization with complete Freund’s adjuvants and two boosters with incomplete Freund’s adjuvant (Fig. 3a). Two weeks after the second IFA boost, we placed eight ticks per mouse which were allowed to feed until repletion, ranging from 3 to 5 days. No difference was found in post-feeding tick weight compared to the ovalbumin group (Fig. 3b), nor did we observe a difference in *B. burgdorferi* migration to the salivary glands or in *B. burgdorferi* transmission to the host by RT-qPCR (Fig. 3c). Based on qPCR analysis, *B. burgdorferi* loads in skin tissue from the tick bite site (ears) as well as deeper tissue were similar between the rISDLP-immunized animals and the ovalbumin-immunized animals (Fig. 3d, e).

### *isdlp* RNAi reduces *B. burgdorferi* migration to the salivary glands and transmission to the murine host

**Fig. 4:**
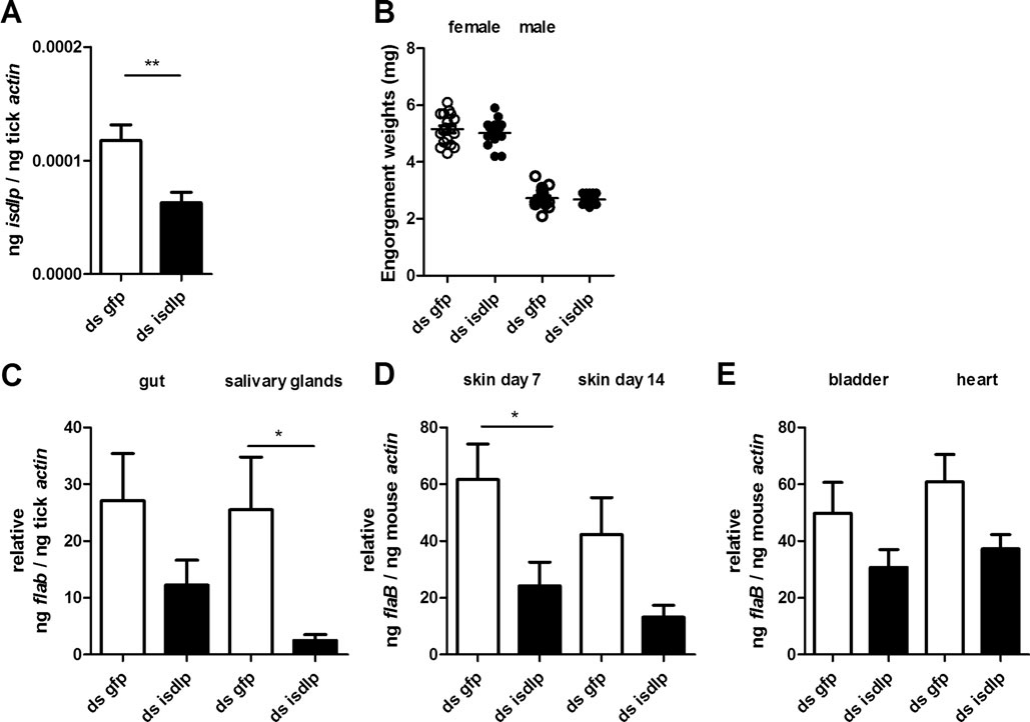
Silencing of ISDLP expression by RNA interference results in decreased *B. burgdorferi* burden in the salivary glands and in murine skin. Double-stranded *isdlp* (ds *isdlp*) or ds *gfp* as a control was injected through the anal pore 3 h prior to *B. burgdorferi*-infected *I. scapularis* challenge (five ticks/mouse, five mice per experiment). Mice were sacrificed after 14 days. **a** RT-qPCR assessment of *isdlp* expression in the gut. **b** Engorgement weights of ticks post-feeding. Each *data point* represents one tick. A tick was considered female when >3.5 mg. **c** *B. burgdorferi* burden in tick guts and salivary glands post-feeding. **d**, **e** qPCR assessment of *B. burgdorferi* burden in the murine skin at 7 days and in the skin, bladder, and heart at 14 days post-tick feeding. The *error bars* represent mean ± SEM, and the mean values that were significantly different in a two-tailed non-parametric Mann–Whitney test are indicated by an *asterisk* (*p* ≤ 0.05). The pooled results of two independent mouse experiments are shown

It is likely that in the active immunization experiment, the function of ISDLP in relation to *B. burgdorferi* migration and transmission remained unaffected by murine anti-ISDLP antibodies present in the gut during tick feeding. To circumvent the use of antibodies to provide an insight in the role of ISDLP during *B. burgdorferi* transmission, we performed another experiment in which *isdlp* expression in ticks was silenced by RNAi. We injected double-stranded (ds) *isdlp* RNA or ds *gfp* RNA as a control in *B. burgdorferi*-infected *I. scapularis* nymphs before placing four to five ticks on C3H/H3N mice. The decrease of *isdlp* expression in the tick gut was confirmed by RT-qPCR (Fig. 4a). No difference in tick engorgement weights was observed between *isdlp* and the control ticks, indicating that reduced *isdlp* expression does not influence successful tick feeding (Fig. 4b). In contrast with the active immunization, significantly lower *B. burgdorferi* loads were detected in the salivary glands (Fig. 4c). Furthermore, qPCR on DNA from skin tissue showed significantly lower *B. burgdorferi* numbers in mice on which *isdlp* silenced ticks fed compared to the control group (Fig. 4d). However, *B. burgdorferi* loads were not significantly different at a later time point (*t* = 14 days) or in deeper tissues such as joint and heart, indicating that *B. burgdorferi* growth in the murine host is not affected by the reduced expression of *isdlp* in the tick (Fig. 4e).

## Discussion

During tick feeding, while the tick gut adapts to cope with the uptake of blood, *B. burgdorferi* becomes metabolically active, replicates, and binds to hypertrophic and differentiating gut cells in order to cross the gut barrier [6, 15]. The molecular mechanisms that direct the growth and migration from the tick gut and entry into salivary glands are only beginning to unfold [5]. *B. burgdorferi* has been shown to bind to host and tick proteins to facilitate its survival and dissemination [16]. To better understand vector–*B. burgdorferi* interactions, we performed a YSD screening to identify *I. scapularis* gut proteins that interact with *B. burgdorferi*. We identified four *B. burgdorferi*-interacting tick proteins of which one, Ixofin3D, has been previously described [12]. Here, we characterize one of the other three proteins, referred to as ISDLP, and assess its role in *B. burgdorferi* transmission.

Computer-based protein structure and function predictions showed that ISDLP is similar to the conserved transmembrane protein dystroglycan. Recombinant ISDLP binds to *B. burgdorferi* and is abundantly expressed on the surface of gut epithelial cells during tick feeding, which was in accordance with previous assessment of ISDLP expression by RT-qPCR [12]. The function of ISDLP for *I. scapularis* has not yet been described. In other organisms, dystroglycan is part of the dystrophin-associated protein complex and is cleaved post-translationally into two subunits, α- and β-dystroglycan, that together form the dystroglycan complex [14]. The dystroglycan complex can bind to the extracellular matrix by binding to laminin. Furthermore, studies have shown that the dystroglycan complex is involved in cell adhesion-mediated signaling, tissue remodeling, and cell polarity and that β-dystroglycan is involved in MAPK signaling [17, 18]. The functional role of ISDLP on cell metabolism, cell signaling, or tissue remodeling during tick feeding remains to be defined.

RNAi-mediated decrease in the expression of ISDLP reduced *B. burgdorferi* transmission to the murine host. While *B. burgdorferi* load in the gut was not altered, *B. burgdorferi* load in the salivary glands was significantly reduced, suggesting that ISDLP might have a role in *B. burgdorferi* migration from the gut. There have been no previous reports on *B. burgdorferi* interactions with human dystroglycan, which is identified as a receptor for a number of viruses as well as for *Mycobacterium leprae* [19, 20]. *B. burgdorferi* is known to bind to extracellular matrix proteins such as decorin and fibronectin among others [15, 21], and we speculate that human dystroglycan could be a ligand for *B. burgdorferi* in humans or other vertebrates. The specific binding partner of *B. burgdorferi* that binds ISDLP and the mechanism by which ISDLP promotes *B. burgdorferi* migration and transmission remain to be understood. Although silencing of ISDLP by RNAi did not impair tick feeding, it cannot be excluded that the effect on *B. burgdorferi* transmission is the result of ISDLP-mediated processes in the feeding gut, e.g., altered gut tissue remodeling or a reduced barrier.

Based on our findings that ISDLP interacts with *B. burgdorferi* and that ISDLP is expressed during tick feeding, we speculated that ISDLP could be a target to impair transmission. Tick gut antigens could be useful to target *B. burgdorferi* migration from the gut and derail transmission early in the process, i.e., “nipping it in the bud.” While RNAi-mediated interference of ISDLP expression decreased *B. burgdorferi* transmission, active immunization against ISDLP did not impair *B. burgdorferi* migration to the salivary glands and did not reduce transmission to the murine host. There are several explanations for the discrepancy between our RNAi experiment and immunization experiment. While RNAi-mediated silencing is initiated prior to and during tick feeding, sufficient antibody uptake by the tick from the host might take more than 24–36 h coincident with the arrival of blood meal into the tick gut [22]. Thus, delayed entry of antibodies might allow *B. burgdorferi* to exploit the gut ISDLP and continue its migration from the gut. In order to prevent *B. burgdorferi* egress from the gut, anti-ISDLP antibodies have to significantly neutralize ISDLP’s interaction with *B. burgdorferi*. Immunofluorescence microscopy suggests that ISDLP is ubiquitously represented on the tick gut. Affinity of the antibody binding as well as amounts of antibody that enter the gut would determine the successful abrogation of *B. burgdorferi*–ISDLP interaction. In addition, other possibilities that impaired a protective effect of anti-ISDLP antibodies could be the inaccessibility of the protective epitope within the tick or the inability of the generated antibodies to block the interaction between ISDLP and *B. burgdorferi*. The latter possibility is supported by in vitro observations that binding of ISDLP to *B. burgdorferi* could not be blocked by anti-ISDLP antibodies (Online Resource 2). More research to identify specific regions of ISDLP that interact with *B. burgdorferi* as well as the *B. burgdorferi* ligand that interacts with ISDLP would be informative for effectively blocking ISDLP–*B. burgdorferi* interaction through antibodies.

While several tick proteins with pharmacological functions critical for tick feeding and *B. burgdorferi* transmission have been identified [23], vaccine targeting has been confounded by the functional and structural paralogy of the tick transcriptome [24]. Recent efforts have increased our understanding of tick–*B. burgdorferi* interactions that facilitate *B. burgdorferi* migration within the tick. Vaccines targeting these *B. burgdorferi*-interacting tick proteins provide another avenue to interrupt *B. burgdorferi* transmission. It is becoming evident that *B. burgdorferi* exploits multiple tick proteins to temporally and spatially orchestrate its migration from the gut, entry into salivary glands, and transmission to the host. Elucidation of *B. burgdorferi*-interacting tick proteins that facilitate the various aspects of transmission would help design an optimal combination of vaccine targets that would provide a synergistic impairment of transmission to the vertebrate host.

## Electronic supplementary material

ESM 1 (DOCX 15 kb)

ESM 2 (DOCX 104 kb)

## References

[1] Steere AC, Coburn J, Glickstein L (2004). The emergence of Lyme disease. J Clin Invest.

[2] de la Fuente J, Estrada-Pena A, Venzal JM, Kocan KM, Sonenshine DE (2008). Overview: ticks as vectors of pathogens that cause disease in humans and animals. Front Biosci.

[3] Estrada-Pena A, Jongejan F (1999). Ticks feeding on humans: a review of records on human-biting Ixodoidea with special reference to pathogen transmission. Exp Appl Acarol.

[4] Pal U, Li X, Wang T, Montgomery RR, Ramamoorthi N, Desilva AM, Bao F, Yang X, Pypaert M, Pradhan D (2004). TROSPA, an Ixodes scapularis receptor for Borrelia burgdorferi. Cell.

[5] Radolf JD, Caimano MJ, Stevenson B, Hu LT (2012). Of ticks, mice and men: understanding the dual-host lifestyle of Lyme disease spirochaetes. Nat Rev Microbiol.

[6] Dunham-Ems SM, Caimano MJ, Pal U, Wolgemuth CW, Eggers CH, Balic A, Radolf JD (2009). Live imaging reveals a biphasic mode of dissemination of Borrelia burgdorferi within ticks. J Clin Invest.

[7] Plotkin SA (2011). Correcting a public health fiasco: the need for a new vaccine against Lyme disease. Clin Infect Dis.

[8] Hovius JW, van Dam AP, Fikrig E (2007). Tick-host-pathogen interactions in Lyme borreliosis. Trends Parasitol.

[9] Schuijt TJ, Coumou J, Narasimhan S, Dai J, Deponte K, Wouters D, Brouwer M, Oei A, Roelofs JJ, van Dam AP (2011). A tick mannose-binding lectin inhibitor interferes with the vertebrate complement cascade to enhance transmission of the Lyme disease agent. Cell Host Microbe.

[10] Dai J, Wang P, Adusumilli S, Booth CJ, Narasimhan S, Anguita J, Fikrig E (2009). Antibodies against a tick protein, Salp15, protect mice from the Lyme disease agent. Cell Host Microbe.

[11] Dai J, Narasimhan S, Zhang L, Liu L, Wang P, Fikrig E (2010). Tick histamine release factor is critical for *Ixodes scapularis* engorgement and transmission of the Lyme disease agent. PLoS Pathog.

[12] Narasimhan S, Coumou J, Schuijt TJ, Boder E, Hovius JW, Fikrig E (2014). A tick gut protein with fibronectin III domains aids Borrelia burgdorferi congregation to the gut during transmission. PLoS Pathog.

[13] Narasimhan S, Perez O, Mootien S, DePonte K, Koski RA, Fikrig E, Ledizet M (2013). Characterization of ixophilin, a thrombin inhibitor from the gut of Ixodes scapularis. PLoS One.

[14] Ibraghimov-Beskrovnaya O, Ervasti JM, Leveille CJ, Slaughter CA, Sernett SW, Campbell KP (1992). Primary structure of dystrophin-associated glycoproteins linking dystrophin to the extracellular matrix. Nature.

[15] Schmit VL, Patton TG, Gilmore RD (2011). Analysis of Borrelia burgdorferi surface proteins as determinants in establishing host cell interactions. Front Microbiol.

[16] Coburn J, Leong J, Chaconas G (2013). Illuminating the roles of the Borrelia burgdorferi adhesins. Trends Microbiol.

[17] Constantin B (2014). Dystrophin complex functions as a scaffold for signalling proteins. Biochim Biophys Acta.

[18] Spence HJ, Dhillon AS, James M, Winder SJ (2004). Dystroglycan, a scaffold for the ERK-MAP kinase cascade. EMBO Rep.

[19] Rambukkana A, Salzer JL, Yurchenco PD, Tuomanen EI (1997). Neural targeting of Mycobacterium leprae mediated by the G domain of the laminin-alpha2 chain. Cell.

[20] Cao W, Henry MD, Borrow P, Yamada H, Elder JH, Ravkov EV, Nichol ST, Compans RW, Campbell KP, Oldstone MB (1998). Identification of alpha-dystroglycan as a receptor for lymphocytic choriomeningitis virus and Lassa fever virus. Science.

[21] Hallstrom T, Haupt K, Kraiczy P, Hortschansky P, Wallich R, Skerka C, Zipfel PF (2010). Complement regulator-acquiring surface protein 1 of Borrelia burgdorferi binds to human bone morphogenic protein 2, several extracellular matrix proteins, and plasminogen. J Infect Dis.

[22] Anderson JF, Magnarelli LA (2008). Biology of ticks. Infect Dis Clin N Am.

[23] Hovius JW, Levi M, Fikrig E (2008). Salivating for knowledge: potential pharmacological agents in tick saliva. PLoS Med.

[24] Ribeiro JM, Alarcon-Chaidez F, Francischetti IM, Mans BJ, Mather TN, Valenzuela JG, Wikel SK (2006). An annotated catalog of salivary gland transcripts from Ixodes scapularis ticks. Insect Biochem Mol Biol.

[25] Schultz J, Milpetz F, Bork P, Ponting CP (1998). SMART, a simple modular architecture research tool: identification of signaling domains. Proc Natl Acad Sci.

[26] Kelley LA, Sternberg MJ (2009). Protein structure prediction on the Web: a case study using the Phyre server. Nat Protoc.

