# Supplementary material for: Ixodes scapularis dystroglycan-like protein promotes Borrelia burgdorferi migration from the gut
Source: J Mol Med (Berl). 2015 Nov 23;94:361–70. doi: 10.1007/s00109-015-1365-0 (PMC4803822; doi:10.1007/s00109-015-1365-0)
Supplement: Supplementary file 1 — (DOCX 15 kb) [file 109_2015_1365_MOESM1_ESM.docx]

***Ixodes scapularis* dystroglycan-like protein promotes *Borrelia burgdorferi* migration from the gut**

**Journal of Molecular Medicine**

Jeroen Coumou^1^, Sukanya Narasimhan^2^, Jos J Trentelman^1^, Alex Wagemakers^1^, Joris Koetsveld^1^, Jasmin I. Ersoz^1^, Anneke Oei^3^, Erol Fikrig^2^, Joppe W. Hovius^1^

1. Center for Experimental and Molecular Medicine, Academic Medical Center, University of Amsterdam, 1105 AZ Amsterdam, the Netherlands.

2. Department of Internal Medicine, Yale University School of Medicine, 06511 New Haven, CT, USA.

3. Department of Medical Microbiology, Academic Medical Center, University of Amsterdam, 1105 AZ Amsterdam, the Netherlands.

**Corresponding author:** J. Coumou, j.coumou@amc.uva.nl

**Table S1. Primers utilized in this study**

| **Primer name** | **Primer sequence** | **Function** |
| --- | --- | --- |
| ISDLP_RTFW | AGGACTCTGCATGGACAAGG | Native *isdlp* expression and ‘5 END RLM RACE |
| ISDLP_RTRV | AGCAGCATCACTGCAATCAC | Native *isdlp* expression |
| ISDLP_309RV | GAAGTTCAGGGTTGCTCCTG | ‘3 END RLM RACE |
| ISDLP_265RV | ACCGAAAAGACGTCCCTAGC | ‘3 END RLM RACE |
| ISDLP_1969FW | GTGATTGCAGTGATGCTGCT | ‘5 END RLM RACE |
| ISDLP_604RV | CGCCAATGTAGTGGGAGTGT | *isdlp* sequencing |
| ISDLP_472FW | CTAAAGCGCCTCGAAGCAT | *isdlp* sequencing |
| ISDLP_1575RV | ACTAACACCTTGCGGCTGAT | *isdlp* sequencing |
| ISDLP_1277FW | GCACCACACGGTACCTAACC | *isdlp* sequencing |
| ISDLP_1FW | CAGGTGGATGCAGTTTGAGT | *isdlp* expression |
| ISDLP_2904RV | AAAAAATCTAGAGTAGATGTCGTCGTCTGC | rISDLP expression |
| ISDLP_dsRNAFW | TAATACGACTCACTATAGGGAGACCACGAACGCAAGAACTGTA | dsRNA *isdlp* |
| ISDLP_dsRNARV | TAATACGACTCACTATAGGGAGAATCGTGTTGACCGTCACGTA | dsRNA *isdlp* |
