# Supplementary material for: Ixodes scapularis dystroglycan-like protein promotes Borrelia burgdorferi migration from the gut
Source: J Mol Med (Berl). 2015 Nov 23;94:361–70. doi: 10.1007/s00109-015-1365-0 (PMC4803822; doi:10.1007/s00109-015-1365-0)
Supplement: Supplementary file 2 — (DOCX 104 kb) [file 109_2015_1365_MOESM2_ESM.docx]

***Ixodes scapularis* dystroglycan-like protein promotes *Borrelia burgdorferi* migration from the gut**

**Journal of Molecular Medicine**

Jeroen Coumou^1^, Sukanya Narasimhan^2^, Jos J Trentelman^1^, Alex Wagemakers^1^, Joris Koetsveld^1^, Jasmin I. Ersoz^1^, Anneke Oei^3^, Erol Fikrig^2^, Joppe W. Hovius^1^

1. Center for Experimental and Molecular Medicine, Academic Medical Center, University of Amsterdam, 1105 AZ Amsterdam, the Netherlands.

2. Department of Internal Medicine, Yale University School of Medicine, 06511 New Haven, CT, USA.

3. Department of Medical Microbiology, Academic Medical Center, University of Amsterdam, 1105 AZ Amsterdam, the Netherlands.

**Corresponding author:** J. Coumou, j.coumou@amc.uva.nl


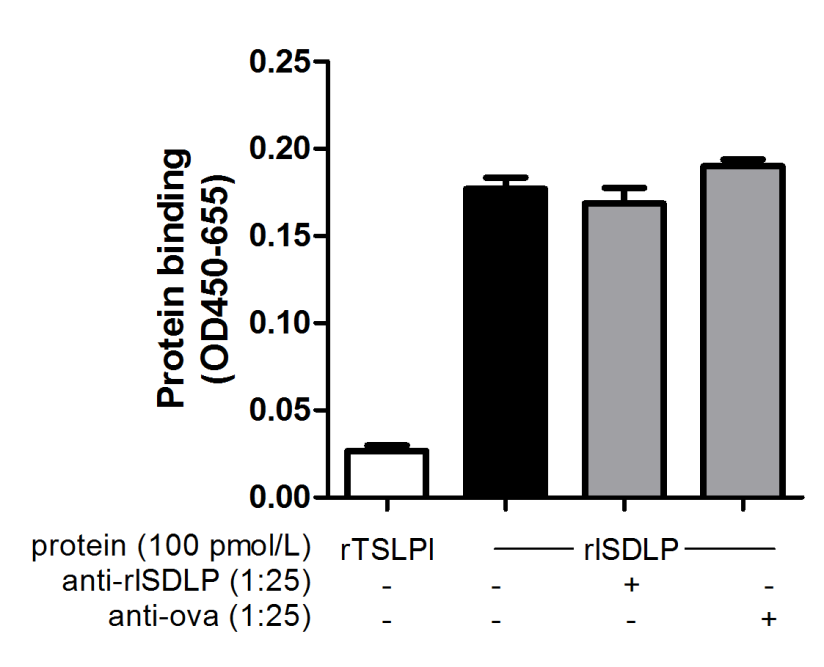


**ESM 2** Anti-ISDLP antibodies do not block binding of rISDLP to *B. burgdorferi*. *B. burgdorferi* membrane extract purified as described in the manuscript was coated (1 µg/ml) on high binding microtiter plates (Microlon, Greiner, Germany) overnight at RT. Wells were blocked with PBS/1% BSA at RT for 1 h and incubated with rTSLPI or rISDLP (100 pmol/ml) diluted in PBS/0.05%Tween20/1% BSA for 1 h. In addition, rISDLP was preincubated with 1:25 anti-ISDLP or anti-ovalbumin rabbit serum for 30 minutes before adding it to the *B. burgdorferi* extracted coated wells. Wells were washed and incubated with 1:5000 diluted mouse anti-V5 HRP IgG. Bound antibody was detecting using TMB as substrate (Thermoscientific, IL)
